# Exploitation of the genetic potential of Thinopyrum and Agropyron genera to protect wheat from diseases and environmental stresses

**DOI:** 10.18699/vjgb-24-60

**Published:** 2024-09

**Authors:** L.Ya. Plotnikova, V.V. Knaub

**Affiliations:** Omsk State Agrarian University named after P.A. Stolypin, Omsk, Russia; Omsk State Agrarian University named after P.A. Stolypin, Omsk, Russia

**Keywords:** wheat breeding, tertiary gene pool, Thinopyrum, Agropyron, introgression, resistance for disease and abiotic stresses, nonhost resistance, durable resistance, селекция пшеницы, третичный генофонд, Thinopyrum, Agropyron, интрогрессия, устойчивостьк болезням и абиотическим стрессам, устойчивость нехозяев, длительная устойчивость

## Abstract

Common wheat is one of the most important food crops in the world. Grain harvests can be increased by reducing losses from diseases and environmental stresses. The tertiary gene pool, including Thinopyrum spp., is a valuable resource for increasing genetic diversity and wheat resistance to fungal diseases and abiotic stresses. Distant hybridization between wheat and Thinopyrum spp. began in the 1920s in Russia, and later continued in different countries. The main results were obtained using the species Th. ponticum and Th. intermedium. Additionally, introgression material was created based on Th. elongatum, Th. bessarabicum, Th. junceiforme, Agropyron cristatum. The results of introgression for resistance to diseases (leaf, stem, and stripe rusts; powdery mildew; Fusarium head blight; and Septoria blotch) and abiotic stresses (drought, extreme temperatures, and salinity) to wheat was reviewed. Approaches to improving the agronomic properties of introgression breeding material (the use of irradiation, ph-mutants and compensating Robertsonian translocations) were described. The experience of long-term use in the world of a number of genes from the tertiary gene pool in protecting wheat from leaf and stem rust was observed. Th. ponticum is a nonhost for Puccinia triticina (Ptr) and P. graminis f. sp. tritici (Pgt) and suppresses the development of rust fungi on the plant surface. Wheat samples with the tall wheatgrass genes Lr19, Lr38, Sr24, Sr25 and Sr26 showed defence mechanisms similar to nonhosts resistance. Their influence led to disruption of the development of surface infection structures and fungal death when trying to penetrate the stomata (prehaustorial resistance or stomatal immunity). Obviously, a change in the chemical properties of fungal surface structures of races virulent to Lr19, Lr24, Sr24, Sr25, and Sr26 leads to a decrease in their adaptability to the environment. This possibly determined the durable resistance of cultivars to leaf and stem rusts in different regions. Alien genes with a similar effect are of interest for breeding cultivars with durable resistance to rust diseases and engineering crops with the help of molecular technologies.

## Abstract (ru)

Мягкая пшеница – одна из важнейших продовольственных культур в мире. Сборы ее зерна можно увеличить, сократив потери от болезней и стрессов окружающей среды. Третичный генофонд, включая виды рода Thinopyrum, является ценным ресурсом для увеличения генетического разнообразия и повышения устойчивости пшеницы к грибным заболеваниям и абиотическим стрессам. Отдаленная гибридизация между пшеницей и Thinopyrum spp. была начата в 1920-х гг. в России и позднее продолжена в разных странах. Основные результаты получены с использованием видов Th. ponticum и Th. intermedium. Дополнительно был создан интрогрессивный материал на основе видов Th. elongatum, Th. bessarabicum, Th. junceiforme, Agropyron cristatum. В статье приведен обзор результатов применения генетического материала видов рода Thinopyrum для повышения устойчивости пшеницы к болезням (бурой, стеблевой и желтой ржавчине, мучнистой росе, фузариозу колоса и септориозным пятнистостям) и абиотическим стрессам (засухе, экстремальным температурам и засолению). Описаны подходы к улучшению агрономических свойств интрогрессивного селекционного материала (применение радиации, ph-мутантов и компенсирующих робертсоновских транслокаций). Проанализирован опыт длительной защиты пшеницы от листовой и стеблевой ржавчины в мире с помощью ряда генов третичного генофонда. Вид Th. ponticum является нехозяином для Puccinia triticina (Ptr) и P. graminis f. sp. tritici (Pgt) и подавляет развитие ржавчинных грибов на поверхности растений. Образцы пшеницы с пырейными генами Lr19, Lr38, Sr24, Sr25 и Sr26 проявляют защитные механизмы, сходные с механизмами нехозяев, что приводит к нарушению развития поверхностных инфекционных структур и гибели грибов при попытке внедрения в устьица (прегаусториальная устойчивость или устьичный иммунитет). Очевидно, изменение химических свойств поверхностных структур рас, вирулентных к Lr19, Lr24, Sr24, Sr25 и Sr26, приводит к снижению их приспособленности к среде, что влияет на длительность устойчивости сортов к ржавчинным болезням. Чужеродные гены с аналогичным эффектом представляют интерес для селекции сортов с длительной устойчивостью к ржавчинным заболеваниям, а также конструирования культуры с помощью молекулярных технологий.

## Introduction

Cultivated wheat species, Triticum aestivum L. and T. durum Desf., are among the most important crops for world nutrition. It is assumed that the world’s population will exceed 9.7 billion people by 2050. To provide nutrition for such a population, it is necessary to increase grain production to 900 million metric tons (Baker et al., 2020; Kumar et al., 2022). Common wheat has high plasticity, allowing it to be cultivated in most agricultural zones of the world. In this regard, wheat grain production has the most significant impact on global food security compared to other cereals (Kuzmanović et al., 2020; Kumar et al., 2022).

During centuries-old wheat breeding, most attention was paid to increasing yield and grain quality. As a result, a significant proportion of the genes determining adaptive capabilities to abiotic and biotic stresses was lost. Stressful environmental conditions and diseases lead to regular and significant losses of grain yield that can reach up to 20–40 % (Curtis, Halford, 2014). An increase in grain yield can be achieved by expanding the crop acreage, increasing the potential productivity by photosynthetic activity, and by reducing losses from abiotic and biotic factors (Savari et al., 2019; FAO Report, 2021). Increasing genetic diversity of wheat is actual for protection crop from diseases and stressful abiotic factors. Thinopyrum and related genera are promising sourсes for enrichment of wheat genetic pool and breeding of cultivars with improved properties.

## Impact of major diseases and abiotic stresses on wheat crops

During the 20th century, large scale wheat monocropping, often homogeneous in resistance to diseases, has been created on different continents. This situation has contributed to pathogen coevolution with plants in agroecosystems increasing over past 70 years (Zhan J.,McDonald, 2013). As a result, the appearance of new pathogens and virulent races within their populations has accelerated, and disease outbreaks have become more frequent (Chen X., 2005; Singh R.P. et al., 2016). The global burden of pathogens and pests on wheat production in 2010–2014 was estimated at 21.5 %, of which 18 % was determined by fungal diseases (Savari et al., 2019). The main losses in the amount of 15.1 % were determined by eight diseases (leaf, stem, and stripe rusts, Septoria tritici blotch and Septoria nodorum blotch, powdery mildew, Fusarium head blight, and tan spot) spread globally. Grain losses vary significantly between world regions, depending on climatic conditions, cultivar heterogeneity, and crop production technologies (McDonald, Stukenbrock, 2016; Singh R.P. et al., 2016).

Abbreviations APR – adult plant resistance ASR – all stage resistance ROS – reactive oxygen species Resistance gene symbols Bdv – barley yellow dwarf virus Fhb – Fusarium head blight Lr – leaf rust Pm – powdery mildew Sr – stem rust Snb – Septoria nodorum blotch Stb – Septoria tritici blotch Wsm – wheat streak mosaic virus Yr – stripe rust

Wheat is affected by leaf, stem, and stripe (yellow) rusts, caused by Puccinia triticina Eriks., P. graminis Pers. f. sp. tritici Eriks. et Henn, and P. striiformis Westend. f. sp. tritici Eriks., respectively. The common features of rust fungi are high reproduction, variability, and airborne dispersal of urediniospores, often over long distances, to new regions and even continents (McDonald, Stukenbrock, 2016; Savari et al., 2019). P. triticina is the most plastic species among wheat rust fungi and regularly affects common wheat crops in many world regions (Kolmer, 2013). In the last decade, leaf rust has increased significantly in the main wheat production regions in China and India (Gao et al., 2019; Aravindh et al., 2020). Wheat stem rust development was suppressed worldwide in the second half of the twentieth century due to the use of cultivars carrying the Sr31 gene transferred from cereal rye, Secale cereale L. (Singh R.P. et al., 2015). However, in Uganda in 1998, the Ug99 race (TTKSK) appeared, which overcame the Sr31 gene, and later other races unrelated to Ug99 (such as Digalu, and Sicilian) appeared. Over two decades, stem rust accelerated in Africa, the Middle East, and in Western Europe (Singh R.P. et al., 2015; Patpour et al., 2022).

Wheat stripe rust used to spread in regions with a cool and humid climate. However, following the appearance of P. striiformis f. sp. tritici clones adapted to high temperatures, there was a rapid spread of the pathogen to new regions. Since the 2000s, stripe rust has become a new threat to grain production in many regions, and regular outbreaks now occur in North and South America, Africa, Northwest Europe, India, China and Russia (Ali S. et al., 2017; Gultyaeva et al., 2022). FAO claims that rusts are the most destructive transborder wheat diseases, making them serious threats to food security worldwide (Singh et al., 2016; FAO Report, 2021).

Another important global wheat disease is powdery mildew, caused by Blumeria graminis f. sp. tritici (DC.) Speer. Previously, powdery mildew, while affecting wheat crops worldwide, prevailed in regions with damp and cool climate. During recent decades, the disease has increased in warmer regions, especially when using intensive technologies with high doses of nitrogenous fertilizers (Zhang R.Q. et al., 2020; Yang G. et al., 2023). Largest crop losses were noted in China, Northwest Europe, and India (Savari et al., 2019).

Septoria blotch diseases are caused by a complex of fungi, the main of them are Zymoseptoria tritici (Roberge ex Desm.) Quaedvl. & Crous. (= Septoria tritici Desm.), and Parastagonospora nodorum (Berk.) Quaedvl. (= Septoria nodorum (Berk.). Significant negative effect of the Septoria fungal complex on wheat crops has been noted since 1980. In previous decades, Septoria tritici blotch caused high grain losses in humid coastal regions of Europe and North America (O’Driscoll et al., 2014; Fones, Gurr, 2015). During the last decade, Septoria tritici blotch has spread to the arid regions of Africa, Northern Kazakhstan, and Western Siberia (Babkenova et al., 2020; Tadesse et al., 2020; Plotnikova et al., 2023b). On the territory of Russia, wheat leaf and ear Septoria diseases are mainly caused by two species – Z. tritici and P. nodorum, and the ratio of pathogens varies significantly depending on the region (Toropova et al., 2020).

Fusarium head blight (FHB) is caused by Fusarium graminearum Schwabe [teleomorph: Gibberella zeae (Schwein.)]. FHB impacts include wheat yield loss, deterioration of grain quality, and mycotoxin contamination, effecting human and animal health (Alisaac, Mahlein, 2023). Frequent FHB epidemics have been occurring since the 1990s in the USA, Canada, South America, China (Zhu et al., 2019; Alisaac, Mahlein, 2023). Tan spot (yellow spot, yellow leaf spot) is caused by the necrotrophic fungus Pyrenophora tritici-repentis (Died.) Dreches [anamorph Drechslera tritici-repentis (Died.) Shoemaker]. The first tan spot epidemics were reported in the 1970s in North America, Australia, and Southern Africa, and later the disease spread globally (Carmona et al., 2006; Phuke et al., 2020).

Climate change is a threat to sustainable crop production. According to the FAO report, the number of disasters (climatological, hydrological, biological and geophysical) per year by decade grew from 90 in 1970s to 360 in 2010s (FAO Report, 2021). The largest increase was noted for weather-related disasters, such as drought, storms, and extreme temperatures. Agriculture is especially vulnerable to increased frequency and intensity of extreme weather-related and climate induced disasters. Damage and loss in agriculture for 2008–2018 was estimated at 63 % (FAO Report, 2021). Among abiotic stresses, drought, extreme temperatures, and soil salinity have the greatest negative impact on wheat grain production (Kosová et al., 2014; Ali N., Mujeeb-Kazi, 2021). About one third of areas most suitable for agriculture, located in the warm regions, are becoming more arid (Goncharov, 2021). Losses in grain production in the world related to a lack of precipitation and extreme temperatures worldwide can reach 28 % (Kumar et al., 2022). Winter varieties produce higher yields compared to spring genotypes. However, to expand winter crops to risky farming areas, it is necessary to improve their winter hardiness (Fisenko, Kuzmina, 2020). The wheat growing area can also be increased by using saline land. However, this requires the breeding of wheat cultivars with high salt tolerance (Yang Z. et al., 2022). The use of a wide range of new genes in breeding is the basis for sustainable defence of bread and durum wheat from stresses (Ceoloni et al., 2014).

## Enhancement of genetic diversity of wheat with alien gene pools

Genetic protection of cultivars is considered the most costeffective and environmentally friendly way to control diseases (Singh R.P. et al., 2016; Gultyaeva et al., 2022). Wild and cultivated cereal species are the main reservoirs of valuable genes for wheat breeding (Ceoloni et al., 2014; Kumar et al., 2022). B. Friebe and co-workers (1996) proposed to divide plant species into primary, secondary, and tertiary gene pools by availability for breeding. Species in primary gene pool have genomes that are homologous to the subgenomes of common wheat (AABBDD), and secondary gene pool has at least one subgenome homologous to wheat. The genetic material of the primary and secondary gene pools can be transferred to common wheat relatively easily in the form of short translocations by recombination of homologous chromosomes

Tertiary gene pool species have genomes that differ from common wheat subgenomes (homoeologous). Introgression of genetic material from tertiary gene pool species to wheat is difficult due to the differences in homoeologous chromosome structure, thus requiring special methods (Friebe et al., 1996; Li H., Wang 2009). The tertiary gene pool includes the genera Aegilops L. (species with U, C, M, T, X genomes), Secale L., Hordeum L., Thinopyrum Á. Löve, Agropyron Gaertn., Leymus Hochst., Haynaldia L. (= Dasypyrum), and Pseudoroegneria (Nevski) A. Löve (Friebe et al., 1996; Kroupin et al., 2019; Kumar et al., 2022). During the 20th century, the most attention in the world was paid to Thinopyrum spp. Perennial Thinopyrum spp. are the components of natural ecosystems and pastures in different Eurasian regions (Tsvelev, 1984; Li H., Wang, 2009). In this regard, they are well adapted to contrasting climatic conditions and have a wide spectrum of resistance genes to abiotic and biotic stresses (Lammer et al., 2005; Li X. et al., 2017).

Scientific research of the genetic potential of Thinopyrum and related genera and the implementation of the results in practice have been ongoing for more than a century (Table 1). The first successful work on distant hybridization between wheat and Thinopyrum spp. (before Agropyron) was carried out by N.V. Tsitsin in Russia during 1920–1930 (Tsitsin, 1979). A set of species was tested, of which two species were most promising for further work, viz. intermediate wheatgrass Thinopyrum intermedium Barkworth &D.R. Dewey and the tall wheatgrass Th. ponticum (Podp.) Z.-W. Liu & R.-C. Wang. It should be noted that the classification of these species has changed many times and is still in the process of formation. In this regard, different names of these species are found in publications of different periods, e. g. Th. intermedium (= Agropyron intermedium (Host) Beauv, Ag. glaucum), and Th. ponticum (= Th. ponticum (Podp.) Barkworth et D.R. Dewey, Ag. elongatum (Host) Beauv, Lophopyrum ponticum (Popd.) A. Löve, Elytrigia pontica (Popd.) Holub.). At the beginning, both wheatgrass species were selected based on perennial habit and tolerance to abiotic stresses (frost resistance, winter hardiness, and drought tolerance). Later, Thinopyrum spp. were recognized as valuable reservoirs of genes for resistance to diseases, and most studies were devoted to this problem (Friebe et al., 1996; Li H., Wang, 2009). At the first stage, the intergeneric amphiploids were obtained, which were crossed with T. aestivum, and later partial amphiploides (named Wheat-Wheatgrass Hybrids, WWHs) with different sets of wheatgrass chromosomes were produced. Some stable WWHs were selected and used as commercial forage regrowing and perennial cultivars (Tsitsin, 1979).

**Table 1.**
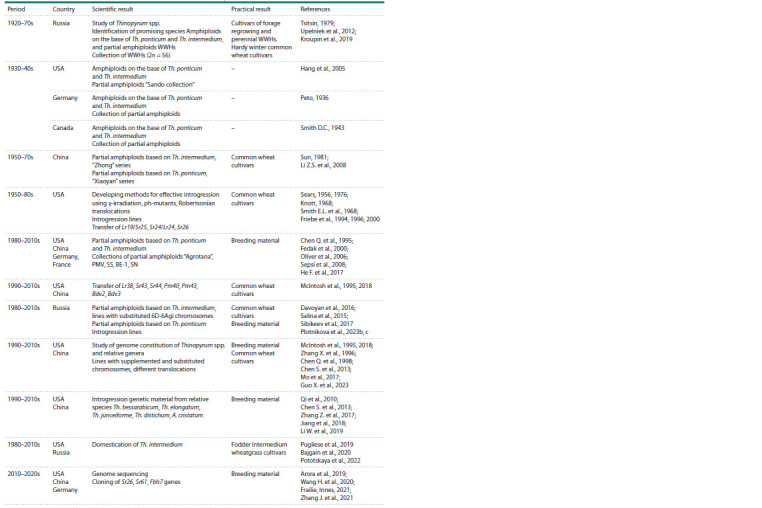
The main stages of introgression of the genetic material from Thinopyrum and related genera to the wheat gene pool Notе. WWHs – wheat-wheatgrass hybrids.

Later, on their basis, winter-hardy wheat cultivars were bred for the European part of Russia (Upelniek et al., 2012). Currently, Tsitsin’s heritage is maintained in the form of octaploid partial amphiploid WWHs collection (2n = 56, including 42 chromosomes of common wheat, and 14 chromosomes from different Thinopyrum’s subgenomes) (Main Botanical Garden of the Russian Academy of Sciences, Moscow, Russia) (Upelniek et al., 2012; Kroupin et al., 2019).

Work on the introgression of the Thinopyrum spp. genetic material into the wheat gene pool was continued in Russia in 1980–2010s. Breeding material on the base of the Avrocum amphiploid was created at the P.P. Lukyanenko National Grain Center (Krasnodar, Russia). Avrocum was obtained by the hybridization of the tetraploid form tetra-Avrora (common wheat cv. Avrora without the D genome) with Th. intermedium (Davoyan et al., 2016). At the same time, amphiploids based on Th. intermedium and later wheat lines with 6D chromosomes substituted by one of the homoeologous chromosomes 6Agi (6Agi or 6Agi2) were obtained. Later, introgression spring common wheat cultivars with alien 6Agi chromosomes were bred at the N.M. Tulaikov Samara Research Institute of Agriculture and at the Federal Centre of Agriculture Research of the South-East Region (Samara and Saratov, accordingly; Russia) for the Volga region (Salina et al., 2015; Sibikeev et al., 2017). New introgression lines of spring common wheat resistant to rusts and Septoria blotch diseases were bred on the basis of Th. ponticum at Omsk State Agrarian University (Omsk, Russia) (Plotnikova et al., 2014, 2021, 2023b).

Introgression of the genetic material of Thinopyrum spp. was carried out independently in different countries of the world (Table 1). In 1930–1940, distant hybridization of T. aestivum with Th. intermedium and Th. ponticum was realized by W.J. Sando in the USA, and a collection of partial amphiploids was obtained (Sando collection, USDA, National Small Grains Germplasm Research Facility, Aberdeen, Idaho, USA) (Hang et al., 2005). Similar work was carried out in Germany and Canada (Peto, 1936; Smith D.C., 1943). Great attention was paid to the introgression of Thinopyrum spp. genetic material in China after the 1950s. S.C. Sun created partial amphi- ploids with Th. intermedium (“Zhong” series), and later, common wheat cultivars were created on their basis (Sun et al., 1981). Z.S. Li produced a set of partial amphiploids on the base of Th. ponticum with resistance to leaf and stripe rusts (e. g. Xiaoyan series, including Xiaoyan 68, Xiaoyan 693, Xiaoyan 7430, Xiaoyan 7631, and Xiaoyan 784) (Li Z.S. et al., 2008). Over time, other series of partial amphiploids were created in different countries: Agrotana (Chen Q. et al., 1995), PMV (Fedak et al., 2000), SS (Oliver et al., 2006), BE-1 (Sepsi et al., 2008), SN (He F. et al., 2017). The partial amphiploids were used for the breeding of numerous supplemented, substituted, and introgression lines with translocations of various size.

The international classification of Thinopyrum spp. is in progress and is being refined using methods of molecular cytogenetics. According to current concepts, the Thinopyrum genus includes species with a wide range of genomes and ploidies (from diploids to decaploids). The diploid accessions (2n = 2x = 14) were identified in Th. elongatum D.R. Dewey and Th. bessarabicum (Savul & Rayss) Á. Löve. The tetraploids (2n = 4x = 28) were determined in Th. junceiforme Á. Löve, and the hexaploids were among Th. intermedium and Th. junceum (2n = 6x = 42). The decaploids were found among Th. ponticum accessions (Chen S. et al., 2013; Mo et al., 2017). Based on cytogenetic and molecular genetic studies, the genomic composition of the hexaploid Th. intermedium is JJJsJsStSt (Chen Q. et al., 1998) or EEEstEstStSt (Wang R.R.- C., 2011), and the decaploid Th. ponticum formula is JJJJJsJsJsJsJsJs (Chen Q. et al., 1998) or EEEbEbExExStStStSt (Zhang X. et al., 1996). The classification of genomes proposed by R.R.-C. Wang (2011) is most often used, but in some articles genome designation J was used. The J or Js subgenomes are highly homologous with the genomes Ee of Th. elongatum and Eb of Th. bessarabicum, accordingly. The St subgenome is closely related to the genome of Pseudoroegneria strigosa (Chen Q. et al., 1998; Wang L. et al., 2017). Agropyron spp. have a different number of P genomes (2n–6n) (Wang R.R.- C., 2011).

## Developing methods for effective introgression of alien genetic material and improving the properties of breeding material

When transferring the genetic material from tertiary gene pool species to wheat, difficulties arise at different stages of the work. Common problems include difficulties in intergeneric crossing, F1 hybrid sterility, and lack of homeologous chromosomes conjugation. As a result of poor conjugation between homoeologous chromosomes, large alien fragments (e. g. whole chromosomes, chromosome arms, or large terminal translocations) are usually transferred into introgression lines (Liu J. et al., 2013; Leonova, 2018). Consequently, the material obtained by distant hybridization is significantly inferior in properties to commercial varieties (Friebe et al., 1996; Li H., Wang et al., 2009). The major reasons for the deterioration of wheat properties is a close linkage between target and undesirable genes (linkage drag), or insufficient genetic complementation between the alien fragment and the wheat genome, and the fact that alien translocation does not compensate the absence of wheat genetic material (Wulff, Moscou, 2014; Hao et al., 2020). In many cases, wheat traits decrease is due to the fact that a large alien fragment does not compensate the loss of important genetic material (Friebe et al., 2005).

In the 1950–1970s, methods were developed that increased the introgression efficiency from relative species to wheat. For the first time, the procedure was implemented when transferring the genetic material from Ae. umbellulata (UU) to common wheat (Sears, 1956). At the first step, an interspecific hybrid was obtained, and its chromosomes were doubled using colchicine. The resulting amphidiploid was backcrossed with wheat, and a substituted line was obtained, which was used as a genetic bridge to transfer leaf rust resistance to common wheat. To facilitate the transfer of genetic material between the U and wheat chromosomes, the pollen of the substituted line was irradiated to induce multiple chromosome breaks followed by recombination of the fragments. As a result of the work, the cv. Transfer was bred, carrying the Lr9 gene for resistance to leaf rust (Sears, 1956).

Later, after the research on wheat meiosis genetic control, it was found that wheat chromosome 5B carries the Ph gene that suppresses the homoeologous chromosomes conjugation. To induce conjugation, it is possible to cross introgression lines with aneuploids in 5B chromosome or to use mutants with the ph genes. The mutant ph1b gene (or similar ph2a and ph2b) facilitates loci exchange between homoeologous chromosomes (Sears, 1976). Similar effects may be supplied by the chromosome 5P of A. cristatum (PPPP) and some accessions of Ae. speltoides (SS) (Friebe et al., 2000; Han et al., 2023).

The hexaploid Th. intermedium crosses relatively easily with common wheat (the average seed setting is about 24 %), and amphiploids can be obtained by direct crossing (Mo et al., 2017). To transfer the genetic material from the decaploid Th. ponticum, amphiploids with the tetraploid wheat T. turgidum (AABB) need to be created (Tsitsin, 1979). Partial am-phiploids with chromosome combinations from various Thinopyrum spp. subgenomes were obtained after backcrossing to common wheat (Friebe et al., 2000; Li H., Wang, 2009). In rare cases, the transfer of homeologous fragment occurs by spontaneous translocation (Knott, 1968). But the induction of translocations using irradiation or induced homeologous recombination with ph1b mutants is more effective for transferring small loci to wheat genome (Sears, 1978). Using these methods, the transfers of at least 134 loci from the Th. ponticum to common wheat were maid (Baker et al., 2020). Recently, a large set of lines with wheatgrass introgressions of various size were bred (Mo et al., 2017; Kroupin et al., 2019).

The transfer of multiple alien fragments and location of introgressions at various chromosomes and their arms is possible (Table 2). For example, Lr38 gene in Th. intermedium derivatives was transferred to four wheat chromosomes (2A, 5A, 3D and 6D) (Friebe et al., 1996). The loci with Sr24/Lr24 genes from the Th. ponticum were identified in two wheat chromosomes (1B and 3D). The loci with Lr19/Sr25 genes were identified in different arms of the 7D chromosome (McIntosh et al., 1995; Friebe et al., 1996). Introgression lines with the best properties were selected for breeding the cultivars.

**Table 2.**
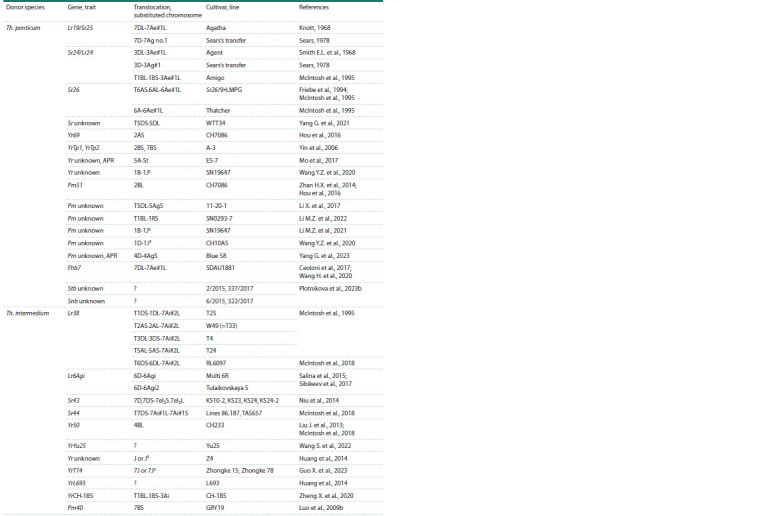
Results of introgression of genetic material from Thinopyrum spp. to wheat gene pool

**Table 2end.**
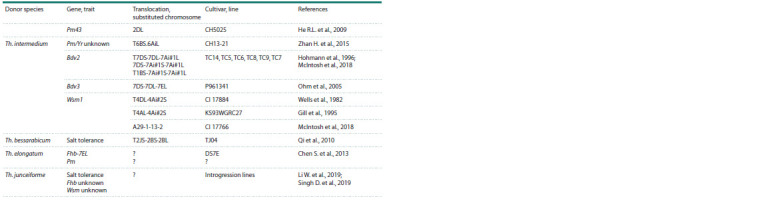
Tab2end.

The improvement of the properties of some introgression lines was achieved by reducing alien fragment size using γ-irradiation of seeds, plants, and pollen, or induced homeologous recombination with ph1b mutants (Sears, 1978). The limited application of Lr19/Sr25 from Th. ponticum in breeding was associated with its linkage with the Y gene determined yellow flour color. Lr19/Sr25 and Y genes were separated using Ph1 deletion lines (Zhang W. et al., 2005). The valuable Fhb7 gene conferring resistance to both Fusarium head blight and Fusarium crown rot was tightly linked with the PSY-E2 gene that determines yellow flour color. Using the ph1b mutant, lines with shortened translocations devoid of the PSY-E2 gene were obtained (Li M.Z. et al., 2022). Thanks to ph1-induced homoeological recombination, interspecific gene transfer from T. aestivum to T. durum was achieved. Three loci with alien Lr19/Sr25/Y and Pm13 genes (from Th. ponticum and Ae. longissima, respectively), and Gli-D1/Glu-D3 (effecting gluten properties) were transferred from common wheat chromosomes 7D, 1D and 3B to durum wheat arms 7AL, 3BS and 1AS (Kuzmanović et al., 2020).

In some cases, noncompensating wheat-alien translocations occur in introgressive material. This is due to the participation in meiosis of homoeologous chromosome arms, which differ in gene sets and their order. Noncompensating translocations provoke genomic duplications or deficiencies, which lead to line genetic instability and prevent the use of a valuable gene in breeding (Friebe et al., 1996). Compensating Robertsonian translocations (RobTs) are used for functional substitution of lost fragments to correct wheat genotypes (Friebe et al., 2005). Such a method was used to improve the properties of the line with the Sr44 gene from Th. intermedium. As a result, the compensating RobT in the form of recombination T7DL×7J#1S was identified, consisting of wheat arm 7DL translocated to the Th. intermediate arm 7J#1S (Liu W. et al., 2013). Similar works were carried out, developing lines with the Sr51, Sr52, and Sr53 genes (from Ae. searsii Feldman & Kislev ex Hammer, and Ae. geniculata Roth, respectively) (Liu W. et al., 2011a, b).

In the rarest cases, substitution of wheat chromosomes by alien ones does not decrease agronomic properties. This was the case for spring common wheat cultivars with chromosome 6D substituted by 6Ai or 6Ai2 from the Th. intermedium J(=E) subgenome. A set of cultivars with the 6Ai or 6Ai2 chromosomes (homologous) were bred in the Russian Volga region (Multi 6R, Belyanka, Voevoda, Lebedushka, Tulaikovskaya 5, Tulaikovskaya 100, Tulaikovskaya Zolotistaya, and others). These cultivars showed broad spectrum resistance to leaf and stem rusts, high yield and grain quality, and drought tolerance (Salina et al., 2015; Sibikeev et al., 2017). In China, the big achievement was the breeding of the cv. Xiaoyan 6 with double translocations from Th. ponticum on the chromosomes 2A and 7D. The cv. Xiaoyan 6 was multi-resistant to fungal diseases, had high yield, grain quality, and environmental plasticity. The cv. Xiaoyan 6 was widely cultivated in the 1980–1990s, and used as a parent for more than 60 common wheat cultivars (Zhang X. et al., 2011).

## Contribution of Th. ponticum and Th. intermedium as sources of useful genes

When introgression of the genetic material of Thinopyrum spp., the main attention was paid to emerging disease challenges, and the studies have become more intensive in recent decades. During 1960–2020, a set of designated resistance genes to leaf, stem, and stripe rusts was transferred from Th. ponticum to T. aestivum. Some of these genes are closely linked and are present in complex translocations, viz. Lr19/ Sr25, Sr24/Lr24, and others are single, viz. Lr29, Sr26, Sr43, Sr61 (=SrB), Yr69 (Table 2) (McIntosh et al., 1995, 2018).

After stem and stripe rusts progressed in 2000s, the cereal species and amphiploid collections were screened for disease resistance. Screening of the five Thinopyrum spp. (242 accessions) showed, that Th. ponticum and Th. intermedium are highly resistant to Ug99 race (Zheng Q. et al., 2014a, b). Partial amphiploids created in China in the 1950s (Xiaoyan 68, Xiaoyan 7430, and Xiaoyan 784) are highly resistant to Ug99 group races (Zheng Q. et al., 2014b). A new introgression line, WTT34, was created, carrying at least one new Sr gene in the T5DS∙5DL translocation (Yang G. et al., 2021). Based on the Xiaoyan 784 amphiploid, the ES-7 line was created with 5A-St substituted chromosomes carrying adult plant resistance (APR) to stripe rust (Mo et al., 2017). Yr69 gene was transferred from the Xiaoyan 7430 amphiploid to wheat chromosome arm 2AS (Hou et al., 2016). In the A-3 line, two putatively new stripe rust resistance genes, YrTp1 and YrTp2, were identified in the chromosome arms 2BS and 7BS, respectively (Yin et al., 2006). Additional undesignated genes were determined in other lines (Zheng Q. et al., 2014a; Wang Y.Z. et al., 2020).

The potential of the genus Thinopyrum is poorly used to protect wheat from powdery mildew. Currently, only Pm51 (among 65 designated genes) has been transferred from Th. ponticum (McIntosh et al., 2018). Pm51 confers broadspectrum all-stage resistance (ASR) to the disease (Zhan H.X. et al., 2014). New unknown Pm genes were identified in lines SN19647 and CH10A5, in which 1B and 1D chromosomes were replaced by 1Js chromosomes (Wang Y.Z. et al., 2020; Li M.Z. et al., 2021). Lines 11-20-1 (with the T5DL∙5AgS translocation) and SN0293-2 showed ASR resistance to a set of races (Li X. et al., 2017; Li M.Z. et al., 2022). In the blue-grained wheat line Blue 58 with a chromosome 4Ag(4D) substitution, a gene(s) for APR was present in the short arm of 4Ag that has determined resistance to powdery mildew for over forty years (Yang G. et al., 2023).

In addition to diseases caused by biotrophic fungi (rusts and powdery mildew), an increase of diseases caused by hemibiotrophic, necrotrophic and viral pathogens has been noted. Septoria blotch diseases cause significant losses in grain yield. Crops are mainly protected by the use of fungicides, and genetic protection is poorly implemented (Fones, Gurr, 2015). Currently, there are no resistance genes to Septoria blotches transferred from Thinopyrum spp. among the identified ones. Resistance to Septoria nodorum blotch, Fusarium head blight and tan spot was revealed in the interspecific hybrid Th. ponticum × Th. intermedium (Oliver et al., 2006). Among introgression lines with genetic material of Th. ponticum bred in Western Siberia, a set of lines highly resistant to Septoria tritici blotch and Septoria nodorum blotch, with unknown genes (Stb and Snb, accordingly), were determined (Plotnikova et al., 2023b). Additionally, from Th. ponticum, some resistance genes were transferred, viz. to Fusarium head blight (Fhb7), common root rot, barley yellow dwarf virus (Bdv), wheat streak mosaic virus (Wsm) (Ceoloni et al., 2017; Kumar et al., 2022).

Tall wheatgrass has also been used as a source of valuable traits for wheat, such as resistance to pre-harvest sprouting (Kocheshkova et al., 2017), blue aleurone layer (Liu L.Q. et al., 2018), frost resistance, winter hardiness (Upelniek et al., 2012), and drought tolerance (Kuzmanović et al., 2016; Plotnikova et al., 2023c).

Thinopyrum intermedium is the source of the rust and powdery mildew resistance genes Lr38, Sr43, Sr44, Yr50, Pm40 and Pm43 (McIntosh et al., 1995, 2018; Friebe et al., 1996; He R.L. et al., 2009; Luo et al., 2009а, b; Liu J. et al., 2013; Niu et al., 2014). New genes Lr6Agi and Sr6Agi were identified in the substitution chromosomes 6Agi and 6Agi2 (Salina et al., 2015; Sibikeev et al., 2017). Lines with stripe rust resistance gene YrYu25 were obtained based on amphiploid TAI7047 (Luo et al., 2009a). Four genes for resistance to stripe rust were identified in the Th. intermedium St subgenome (chromosomes 1St, 2St, 3St, and 7St) (Wang S. et al., 2022), and one gene was determined in subgenome J or Js (in the short arm of the supplemented chromosome of line Z4) (Lang et al., 2018). Lines Zhongke 78 and Zhongke 15 with the YrT14 gene in translocations from the alien 7J or 7Js chromosome were bred in China (Guo X. et al., 2023). The YrL693 gene was reported in introgression line L693 (Huang et al., 2014). Potentially new genes for resistance to stripe rust (YrCH-1BS) and powdery mildew were determined in lines with T1BL.1BS-3Ai and T6BS.6AiL translocations (Zhan H. et al., 2015; Zheng X. et al., 2020). Th. intermedium was also a good source of resistance genes to barley yellow dwarf virus (Bdv2, Bdv3), and wheat streak mosaic virus (Wsm1) (Wells et al., 1982; Gill et al., 1995; Hohmann et al., 1996; Ohm et al., 2005; Li H., Wang 2009; McIntosh et al., 2018).

Thinopyrum intermedium can be used not only as a reservoir of genes for improving the food common wheat, but also as a pasture and forage crop. In the 1980s, the work began on domestication of intermedium wheatgrass (Bajgain et al., 2020; Pototskaya et al., 2022). For forage crops, some features are valuable, e. g., perennial habit, rapid regrowth after cutting or grazing, resistance to frost and diseases, and improved feed quality (Hassani et al., 2000; Lammer et al., 2005). As a result of long-term work, perennial intermediate wheatgrass cultivars (Kernza, MN-Clearwater, Sova) were bred for fodder grain and dual-use (for grain and hay) (Hassani et al., 2000; Bajgain et al., 2020; Pototskaya et al., 2022). These cultivars are of interest as gene reservoir for the breeding of wheat cultivars for various purposes.

## Introgression of genetic material of diploid and tetraploid Thinopyrum spp. and relative species to wheat gene pool

Despite great achievements in distant hybridization, introgression from polyploid heterogenomic species is a complex problem. In this regard, diploid and tetraploid species with genomes similar to the Th. intermedium and Th. ponticum subgenomes were used as additional reservoirs of valuable genes, viz. Th. bessarabicum, Th. elongatum, and Th. junceiforme

Th. bessarabicum (JJ or EbEb) showed a high level of salt tolerance (Gorham et al., 1986). To facilitate the gene transfer from Th. bessarabicum, hexaploid and octaploid amphi-ploids (2n = 4x = 42, AABBJJ = AABBEbEb, or 2n = 8x = 56, AABBDDJJ) were created (Qi et al., 2010). On their basis, lines with 5A and 5D chromosomes substituted by 5J were produced. Later, a line with translocation T2JS-2BS∙2BL from the Th. bessarabicum was obtained (Table 2) (Guo J. et al., 2016).

The genomic composition of Th. elongatum is currently being clarified using methods of molecular cytogenetics, and di-, tetra-, hexa-, and decaploid forms with the E genome were identified in it (Colmer et al., 2006; Chen S. et al., 2013; Chen C. et al., 2023; Shi et al., 2023). However, when studying decaploids using differentiating GISH subgenomes with Pseudoroegneria (St) labeled DNA, two St-like and three E-like subgenomes were revealed (Wang L. et al., 2017; Baker et al., 2020). In this regard, the decaploid forms probably belong to Th. ponticum. Th. elongatum has tolerance to salinity, drought, water logging, and extreme temperatures (Li Z.S. et al., 2008; Ceoloni et al., 2014; Li X. et al., 2017; Yang Z. et al., 2022). Diploid and tetraploid accessions were used in hybridization, and lines with supplemented, substituted chromosomes and translocations of different size were obtained. Lines with the short arm of Th. elongatum chromosome 4Ag carry Pm locus for broad-spectrum resistance to powdery mildew (Yang G. et al., 2023).

Tetraploid sea wheatgrass Th. junceiforme (2n = 4x = 28, J1J1J2J2) is adapted to the coastal areas and is characterized by high tolerance to waterlogging, salinity, manganese toxicity, low nitrogen, and heat stress (Singh D. et al., 2019). At first stage, an amphiploid on the base of the Th. junceiforme was obtained, and then the supplemented and introgression lines with translocations were selected. These lines, in addition to abiotic stress tolerance, showed high resistance to Fusarium head blight and wheat streak mosaic virus (Singh D. et al., 2019).

In addition to Thinopyrum spp., the work has been carried out with genus Agropyron. Tetraploid species A. cristatum (2n = 4x = 28, PPPP) is resistant to powdery mildew, stripe and leaf rusts. Introgression lines with translocations from 2P, 5P, 6P, and 7P chromosomes of A. cristatum with valuable genes were produced (Zhang Z. et al., 2017; Jiang et al., 2018). Alien fragments from chromosome 2P and 6P determine a compact plant type with high spike length, spikelet number, and 1,000 grain weight (Zhang Z. et al., 2017; Xu S. et al., 2023). The fragment of a 5P chromosome induced multiple structural rearrangements, including translocations between chromosomes of different subgenomes. This property can potentially be used as a new tool for inducing wheat–alien chromosome recombination (Li W. et al., 2019).

Currently, information is accumulating that species with relative subgenomes may have similar resistance genes. An accession of diploid Th. elongatum has a Fusarium head blight resistance gene (Fhb-7EL) similar to the designated Fhb7 gene from Th. ponticum (accession el2), and in both accessions the Fhb genes were linked with the known Lr19 gene (Ceoloni et al., 2017; Ma et al., 2018; Kuzmanović et al., 2020). These facts emphasize the need for careful study and comparison of introgressive material obtained on the basis of Thinopyrum spp. for determining new genes for resistance to stresses

## Experience of long-term use of tertiary gene pool for defence wheat from diseases

Currently, more than 100 resistance genes to each of the wheat rusts and powdery mildew have been identified, including designated, unknown new genes, and quantitative trait loci (QTLs) (McIntosh et al., 2018). Most of the resistance genes were overcome rather quickly as a result of evolutionary processes in pathogen populations (Kolmer, 2013; Patpour et al., 2022). The use of the tertiary gene pool began in the 1960s, as suitable donor lines were developed. Despite the large amount of introgressive material, a small number of alien genes were intensively used in world breeding programs. This situation was due to the fact that some genes had low protective effect, and others significantly decreased the agronomic properties of cultivars (Friebe et al., 2000). Thus, some of the designated genes transferred from Thinopyrum spp. (viz. Lr29, Lr38, Sr43) were not successfully used in breeding due to negative effects on agronomic traits (Zhang W. et al., 2005).

The experience of intensive use of alien genes over several decades has given us knowledge about their effectiveness and impact on pathogen populations. R. Johnson (1983), based on the analysis of crop production experience, proposed the concept of “durable resistance”, as resistance that remained effective for a long period when a cultivar is deployed over an extensive area and in environments favourable for the disease (Johnson, 1983). One of the most significant achievements in the use of the tertiary gene pool was cultivar breeding with the 1BL/1RS translocation from rye cv. Petkus, carrying genes for resistance to rusts and powdery mildew (Lr26/Sr31/Yr9/ Pm8). The wide spread of the Sr31-protected cultivars led to suppression of the P. graminis f. sp. tritici populations worldwide for several decades, until the appearance of race Ug99 in Uganda in 1998 (Singh R.P. et al., 2015). As a consequence of the spread of races of Ug99 group, Sr31 gene became ineffective in Africa and the Middle East (Singh R.P. et al., 2015; Patpour et al., 2022), but remains effective in the USA, Canada, India, China, and Russia (Brar et al., 2019; Skolotneva et al., 2023; Wu et al., 2023). The history of exploiting cultivars with Sr31 gene shows that it provided durable wheat resistance to stem rust.

various regions of the world has shown that 12 genes turned out to be the most valuable for protection against progressive stem rust. Of these, three genes were obtained from common wheat (Sr2, Sr23 and SrTmp), and two genes were transferred from the primary gene pool (Sr33 and Sr45). The remaining Sr genes were transferred from the tertiary and secondary gene pools, mainly as part of complex loci, viz. from Th. ponticum (Sr24/Lr24, Lr19/Sr25), S. cereale (Sr31/ Lr26/Yr9/Pm8, Sr1RSAmigo/Pm17 and Sr50), T. timopheevii (Sr36/Pm6), Ae. ventricosa (Sr38/Lr37/Yr17) (Singh R.P. et al., 2015). Taking into account the high risk of spreading of Ug99 group races, much attention was paid to the effectiveness of known Sr genes against it. The genes Sr25, Sr26, Sr43, Sr61 from Th. ponticum, as well as Sr44 from Th. intermedium, are effective against the races of Ug99 group (Zhang J. et al., 2021; Pathotype Tracker, 2023). Before the appearance of Ug99 race, virulence to Sr24 was rare in P. graminis f. sp. tritici populations worldwide, but by 2006, virulence appeared in five Ug99 races in Africa (Jin et al., 2008; Bhavani et al., 2019). In Australia, the Sr24 gene has been effective for about 20 years, and Sr26 has remained effective for several decades, which can be considered as long-term resistance to stem rust (Zhang J. et al., 2021).

In the 1983–2012 period, about 12.5 thousand varieties and lines of common wheat were created in the world. The genetic material of Thinopyrum spp., mainly Th. ponticum (93 %), was actively used to protect wheat (Martynov et al., 2016). The distribution of known wheatgrass genes in cultivars varied significantly by region. This may be determined by cultivar adaptation to regional climate, technological requirements for product quality, and pathogen populations. More than half of the North American cultivars had introgressions from Th. ponticum, less often they were present in cultivars in Australia (12.6 %), Asia (14.8 %) and South America (8.5 %) (Martynov et al., 2016). In the USA, most winter varieties were protected by Lr24/Sr24 (Kolmer, 2007), and Lr19/Sr25 was present in 12 % of cultivars. In Australia, Lr24/Sr24 were mainly used to protect wheat from rusts (82 %), and Sr26 and Lr19/Sr25 were used less often. In South Africa and Egypt, about 5 % carried the Lr24/Sr24 genes, and in Russia and China, mainly the Lr19/Sr25 translocation was used (Martynov et al., 2016; Xu X. et al., 2018; Gultyaeva et al., 2021). Over time, the resistance of cultivars with wheatgrass introgressions was overcome by rust fungi in some regions. The Lr19 gene was overcome in Mexico and India (Huerta-Espino, Singh, 1994; Bhardwaj et al., 2005). P. triticina races virulent to Lr24 appeared in North and South America, and South Africa, where translocation Sr24/Lr24 was used for a long time (Park et al., 2002; Kolmer et al., 2007; Li H., Wang, 2009). On the examples of P. triticina populations in the USA, it was shown that the frequencies of virulent to Lr19 and Lr24 alleles were higher in the regions where cultivars with complementary genes were mainly cultivated (Kolmer et al., 2007; Kolmer, 2013). In other world regions, where the Sr24/Lr24 translocation has not been used intensively in breeding, cultivars protected with Sr24 and Lr24 remain resistant to stem and leaf rusts (Xu X. et al., 2018, Gultyaeva et al., 2021).

Common wheat crops in Russia are an interesting model for evaluating the effectiveness of resistance genes to leaf and stem rusts. The major cropping areas are located in the European (North Caucasian, Central Black Earth, Central, and the Volga regions), and in the Asian (South Ural and Western Siberia) parts of the country. Different European and Asian populations of P. triticina and P. graminis f. sp. tritici exist on these crops (Gultyaeva et al., 2021; Skolotneva et al., 2023). The Volga region and the Southern Urals are zones of contact between them, due to the spore transfer with air flows (Gultyaeva et al., 2021).

In 1970–2020, regional cultivars with different Lr and Sr genes from the tertiary gene pool were bred. Some cultivars (from 15 to 30 % in different years) in the Volga region included translocations Lr19/Sr25 and Lr6Agi/Sr6Agi (Sibikeev et al., 2017; Gultyaeva et al., 2021). Lr9 and LrSp genes (from Ae. umbellulata, and Ae. speltoides, respectively) were present in South Ural cultivars, and Lr9 was introduced into West Siberian cultivars. Lr26/Sr31 genes, as well as combinations of less effective Lr and Sr genes, were present in all regions, and Sr24/Lr24 were rare (less than 1 % of cultivars) (Gultyaeva et al., 2021; Baranova et al., 2023). A long-term study of P. triticina populations showed that virulence to Lr19 prevailed in the population of the Volga region until 2010, but as the spectrum of resistance genes expanded, the frequency of alleles decreased. Virulent to Lr19 or Lr9 alleles did not accumulate in P. triticina populations if cultivars with different genes were cultivated in the regions. So, in the Central and Northwest regions, close to the Volga region, virulence to Lr19 and Lr9 was rare in 2001–2010, and disappeared after 2010 (Gultyaeva et al., 2023). In the South Ural and West Siberian regions, Lr9 gene was overcome in 2008 (Meshkova et al., 2012), but Lr19 gene remains effective (Gultyaeva et al., 2021). In all populations, virulence to Lr24 was extremely rare and virulence to Lr6Agi and LrSp was completely absent. There were also no pathotypes virulent to the combinations of Lr19+Lr26 and Lr9+Lr26 (Gultyaeva et al., 2021).

For a long period, stem rust did not significantly affect wheat crops in most regions of Russia. The first strong disease outbreaks were noted in the Volga region in 2013 and 2014, and in Western Siberia and neighbouring Northern Kazakhstan in 2015 (Shamanin et al., 2016; Sibikeev et al., 2016). At the same time, cultivars with Sr31 gene were damaged in both regions (Sibikeev et al., 2016; Plotnikova et al., 2022), but virulent races did not belong to Ug99 group (Patpour et al., 2022). In the following years, virulent pathotypes disappeared from populations, and Sr31 gene remains effective in Russia (Baranova et al., 2023; Skolotneva et al., 2023). By the end of the epidemic of stem rust in Western Siberia in 2015, cultivars and lines with Sr24, Sr25 and Sr26 genes showed moderate susceptibility, but later their resistance was restored (Plotnikova et al., 2023a). In the Volga region, the lines with Sr25 were susceptible to stem rust in 2022, whereas those with Sr24 and Sr26 genes remained highly resistant (Baranova et al., 2023).

After the appearance of virulent pathotypes to single resistance genes, cultivars began to be protected by gene combinations. Combinations of wheatgrass genes (Sr24/Lr24 or Lr19/Sr25) with rye Lr26/Sr31 or T. timopheevii’s Sr36/Pm6 were highly effective against the rusts in different regions of the world (Park et al., 2002, Martynov et al., 2016; Gultyaeva et al., 2021). In the Volga region, the combinations Lr19/ Sr25 + Lr6Agi/Sr6Agi or Lr19/Sr25 + Sr22 (from T. monococcum) were effective (Sibikeev et al., 2017, 2021). Also, high resistance to leaf and stem rusts was demonstrated by cultivars with combinations of translocations Sr24/Lr24 or Lr19/Sr25 with any APR genes present in complex loci, viz. Lr34/Sr57/ Yr18/Pm38, Sr2/Lr27/Yr30, Lr46/Sr58/Yr29, Lr67/Sr55/Yr46 (Aravindh et al., 2020).

## Fitness costs of virulence to genes from tertiary and secondary gene pools and effects of nonhost resistance in introgression wheat

Соevolution of pathogens with host plants is constantly taking place in agroecosystems, aimed at overcoming resistance. Using the example of P. triticina, it was shown that new pathotypes regularly appear in populations, but more than half of them occur once, and then disappear (Gultyaeva et al., 2023). To gain a foothold in populations, new forms need to acquire a set of traits that determine their fitness. Parasitic fitness is defined as the relative ability of a parasitic genotype or population to persist over time and contribute to the future gene pool. Fitness depends on genotype viability and reproductive capability (Park et al., 2002). Virulence contributes to the expansion of the range of affected plants, but may have different fitness costs for pathogens. Under favourable conditions, a new pathotype can accumulate additional modifier genes that increase its fitness. However, under stressful conditions, new genes can lead to a decrease in viability and reproduction, which manifests as a fitness penalty for the parasite (Antonovics, Alexander, 1989; Zhan J., McDonald, 2013).

Plants play the role of habitat for parasitic fungi, which is why cultivar genotypes and crop diversity have a great influence on fungal populations. Fitness cost correlates with durable cultivar resistance to fungal diseases. The suppression of P. graminis f. sp. tritici populations in most world regions after the spread of cultivars protected by the Sr31 gene during 1960–1990, as well as the disappearance of virulent clones to Sr31 from Russian populations in the 2020s, indicates that virulence to this gene dramatically reduces pathogen fitness. At the same time, the appearance of Ug99 race demonstrated the possibility of improving fitness when adapting to wheat cultivars with Sr31 gene in African conditions. The increased frequency of virulent races to Lr19, Lr24, Sr24, and Sr25 in the regions with a significant proportion of cultivars protected by complementary wheatgrass genes and lower concentration in other regions (Kolmer, 2013; Gultyaeva et al., 2021; Baranova et al., 2023) show that the pathotypes gained a competitive advantage on such cultivars, but had fitness penalties of different degrees on other genotypes. Consequently, virulence to Lr28 and LrSp has not been detected in Russian P. triticina populations for decades (Gultyaeva et al., 2021). There was an outbreak of virulence to Lr47 (frequency up to 70 %) in Western Siberia in 2015, but in the following years virulent clones rapidly disappeared from the population (Plotnikova et al., 2018). It is possible that virulence to Lr47 has a high fitness penalty for the pathogen.

Pathogenic fungi are not able to exist on species for which they have not been specialized, so-called nonhosts. Nonhost resistance (NHR) is rarely overcome, so its genetic control and protective mechanisms are of great interest (Niks, 2014). For breeding varieties with durable resistance to diseases, it is considered promising to transfer the defense mechanisms of nonhost species into crops. According to the widely accepted hypothesis formulated in 2010s, nonhost and host resistance is controlled by different genetic systems (PTI and ETI, respectively) (Peng et al., 2018).

When studying the interaction of P. graminis f. sp. tritici with nonhost S. cereale and Th. ponticum, it was found that pathogen development was disrupted at an early stage. This was manifested in the disorientation of fungal infection structures on plant surface, and in suppression of appressoria formation, necessary for penetration into the stomata (Plotnikova et al., 2022, 2023a). When infecting wheat lines and cultivars with introgressed rye and wheatgrass genes (Sr31, Sr24, Sr25, and Sr26), similar signs of the violation of surface fungal structures was revealed. In addition, the generation of reactive oxygen species (ROS) by stomatal guard cells upon contact with the appressoria was revealed in the lines with these genes. ROS generation led to the death of rust fungus before penetration into plant tissues (Plotnikova et al., 2022, 2023a).

Analogous defence mechanisms were established during the interaction of P. triticina with nonhost species, and wheat lines with wheatgrass Lr19 and Lr38 genes (Plotnikova, 2008, 2009). Similar ROS generation by stomatal guard cells, called “stomatal immunity”, was found when Arabidopsis thaliana was infected with non-pathogenic bacteria Escherichia coli and Pseudomonas syringae pv. tomato (Zeng, He, 2010; Melotto, 2017). This indicates that single genes of the secondary and tertiary gene pools may supply defence mechanisms similar to the nonhost ones, which stop infection at the early stages and prevent penetration into the tissues. When virulence occurs, the chemical composition and immunological properties of the fungal cell wall can be changed. Probably, such changes reduce the viability of mutant clones, which leads to a fitness penalty and their disappearance from populations. The appearance of virulence to two genes in the genotype (to Sr24 + Sr31, or Lr19 + Lr26, etc.) leads to the loss/change of a set of important components, which might be lethal for pathotypes. This may explain high cultivar resistance with combinations of wheatgrass and rye translocations to stem and leaf rusts in different world regions.

Thanks to progress in the field of molecular genetics, it has become possible to transfer a set of resistance genes in the form of cassettes (up to five genes) to varieties. The genes controlling PTI-type (non-host) resistance are of particular interest for construction of cultivars with durable resistance to biotrophic pathogens (Liu X. et al., 2021). In this regard, the genes of Thinopyrum and related genera, providing protection similar to nonhosts, are promising for creating effective gene cassettes.

## Conclusion

Increasing the production of wheat grain is a strategic task to provide food for the growing world population. For sustainable grain production, it is necessary to increase the genetic diversity of cultivars. Species of the secondary and tertiary gene pools with homoeological genomes are of great value for crop protection. Thinopyrum and related genera are reservoirs of resistance genes to wheat diseases and abiotic stresses. The most valuable species for breeding are polyploids Th. ponticum and Th. intermedium. Recently, it has been shown that relative species Th. elongatum, Th. bessarabicum, Th. junceiforme, and A. cristatum are also potential donors of valuable traits for wheat improvement. Currently, a large number of introgression lines resistant to a range of wheat diseases (including leaf, stem, and stripe rusts, and powdery mildew, etc.) and tolerant to abiotic factors (such as drought, salinity, and extreme temperature, etc.) have been produced. However, only a small number of introgressions were used in wheat breeding, due to negative effects on agronomic traits. To improve line properties, the work was carried out to reduce the sizes of loci or to use compensating Robertsonian translocations (RobTs).

The experience of long-term cultivation of varieties with the genes from S. cereale and Th. ponticum has shown that they significantly influence P. triticina and P. graminis f. sp. tritici populations. Obviously, virulent alleles to tall wheatgrass and rye genes reduce the fitness of rust fungi, which leads to partial or complete pathotypes elimination from fungal populations. Cultivars with combinations of wheatgrass and rye translocations showed high resistance to leaf and stem rusts in different regions of the world. Th. ponticum and S. cereale are nonhosts to P. graminis f. sp. tritici and P. triticina, and their resistance leads to disruption of the development of fungal structures at the plant surface or when trying to penetrate into the stomata. The introgressed Sr24, Sr25, Sr26, Lr19, Lr38, and Sr31 genes control manifestations of protective mechanisms similar to nonhost resistance. Such action makes these genes (and the genes with analogous action) promising for engineering crops with the help of molecular technologies

## Conflict of interest

The authors declare no conflict of interest.
